# Complete chloroplast genome sequence of *Ligustrum quihoui* (Oleaceae): genome structure and genomic resources

**DOI:** 10.1080/23802359.2019.1687349

**Published:** 2019-11-13

**Authors:** Lei Wang, Ning Wang, Jiahui Sun

**Affiliations:** a Foresty College, Henan University of Science and Technology, Luoyang, China;; b State Key Laboratory Breeding Base of Dao-di Herbs, National Resource Center for Chinese Materia Medica, China Academy of Chinese Medical Sciences, Beijing, China

**Keywords:** Chloroplast genome, *Ligustrum quihoui*, genomic resources, SSR

## Abstract

*Ligustrum quihoui* is popular as landscape plants used as hedges in gardens. In this study, we sequenced the complete chloroplast genome of *L. quihoui* based on next-generation sequencing and used the data to assess genomic resources. The chloroplast genome of *L. quihoui* is 163,575 bp in length consisting of large and small single-copy regions of length 88,072 and 11,493 bp, separated by two IR regions of 32,005 bp. *De novo* assembly and annotation showed the presence of 115 unique genes with 81 protein-coding genes, 30 tRNA genes, and four rRNA genes. A total of 62 perfect chloroplast simple sequence repeats were analyzed in the *L. quihoui*. A maximum-likelihood phylogenomic analysis showed that *L. quihoui* was sister to *L. gracile.*

*Ligustrum quihoui* is an evergreen shrub with attractive and glossy dark green leaves, and popular as landscape plants used as hedges in gardens. The genus *Ligustrum* contains 37–50 species, mostly native to Asia. *Ligustrum* species have long been cultivated widely as hedge plants and are used as medicinal plants in China (Gu et al. 2011). It is necessary to develop genomic resources for *L. quihoui* to provide intragenic information for its utilization and to provide valuable information about the course of evolution of the genus. Chloroplast polymorphism in chloroplast genome has been used for resolving phylogenetic, genetic diversity evaluation, and plant molecular identification (Dong et al. 2012, 2016). In this study, we assembled the chloroplast genome of *L. quihoui* based on Illumina sequencing technology, and retrieved valuable chloroplast genomic resources for this species.

Sample of *L. quihoui* was collected from Shiqian, Guizhou province of China (27°20′12.78″N, 108°09′18.70″E). The specimen was deposited in PE (01342404). Total genomic DNA was extracted and purified following the method of Li et al. (2013). After the construction of shotgun library, high-throughput sequencing was conducted on the Illumina Hiseq X-Ten sequencing platform. The paired-end reads were qualitatively assessed and assembled with SPAdes 3.6.1 (Bankevich et al. 2012). The annotation was performed with Plann (Huang and Cronk 2015). The annotated genomic sequence was submitted to GenBank with the accession number MN510462.

The complete chloroplast genome of *L. quihoui* is 163,575 bp in size, with a pair of IR regions of 32,005 bp that separate a LSC region of 88,072 bp and a SSC region of 11,493 bp. The GC content was 37.9%. The genome consisted of 115 different coding genes, including 81 were protein-coding genes, 30 were distinct tRNA genes, and 4 were rRNA genes. Simple sequence repeats in the *L. quihoui* chloroplast genomes were detected using GMAT (Wang and Wang 2016) with the minimal repeat number set to 10, 5, 4, 3, 3, and 3 for mono-, di-, tri-, tetra-, penta-, and hexanucleotide sequences, respectively. A total of 62 perfect cp microsatellites were analyzed in the *L. quihoui*. The majority of the SSRs in this chloroplast genome were mononucleotides (62.90%) and almost all of the mononucleotides (94.87%) are composed of A/T. Furthermore, there were six di-, nine tri-, two tetra-, two penta-, and four hexanucleotide repeats in the *L. quihoui* chloroplast genome.

Phylogenetic analyses were performed using maximum likelihood (ML) in IQ-TREE (Nguyen et al. 2015) with the best best-fit model selected by ModelFinder (Kalyaanamoorthy et al. 2017) using the concatenated coding sequences of 82 chloroplast-coding genes for a group of Oleaceae species. Bootstrap values were calculated using the in-built UFBoot within IQ-TREE. The reconstructed phylogeny revealed that *L. quihoui* was sister to *L. gracile* (Figure 1). The whole chloroplast genome sequences provided sufficient genetic information for species identification and phylogenetic reconstruction of the genus *Ligustrum*.

**Figure 1.**
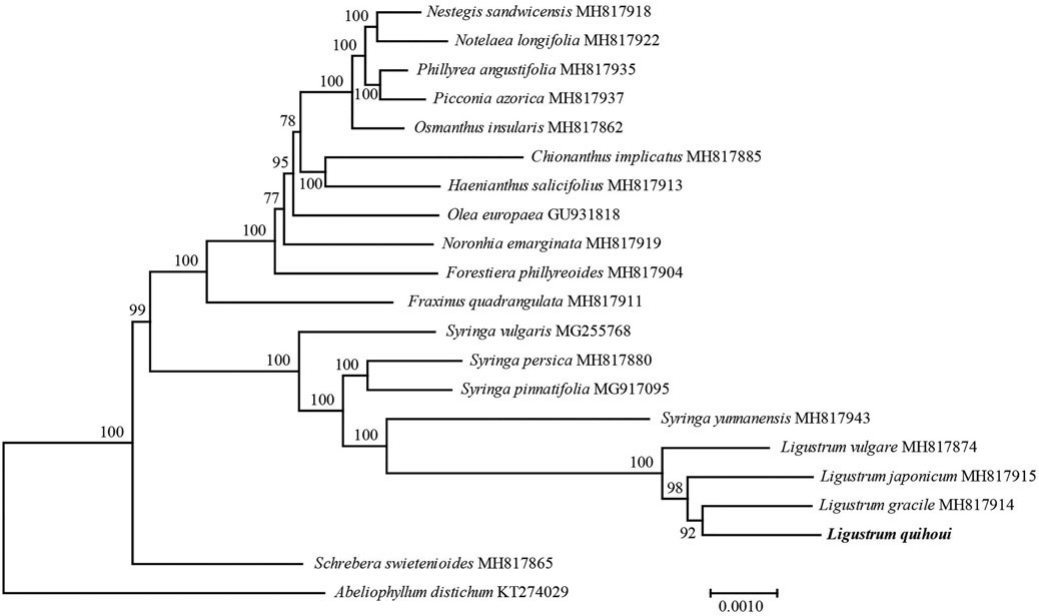
Phylogenetic tree reconstruction of 21 taxa using maximum likelihood (ML) methods based on 82 genes in the chloroplast genome sequences. ML bootstrap support value presented at each node.
